# Structural Characteristics of Novel Protein Folds

**DOI:** 10.1371/journal.pcbi.1000750

**Published:** 2010-04-22

**Authors:** Narcis Fernandez-Fuentes, Joseph M. Dybas, Andras Fiser

**Affiliations:** 1 University of Leeds, Leeds Institute of Molecular Medicine Section of Experimental Therapeutics, St. James's University Hospital, Leeds, United Kingdom; 2 Department of Systems and Computational Biology, Department of Biochemistry, Albert Einstein College of Medicine, Bronx, New York, United States of America; National Cancer Institute, United States of America and Tel Aviv University, Israel

## Abstract

Folds are the basic building blocks of protein structures. Understanding the emergence of novel protein folds is an important step towards understanding the rules governing the evolution of protein structure and function and for developing tools for protein structure modeling and design. We explored the frequency of occurrences of an exhaustively classified library of supersecondary structural elements (*Smotifs*), in protein structures, in order to identify features that would define a fold as novel compared to previously known structures. We found that a surprisingly small set of Smotifs is sufficient to describe all known folds. Furthermore, novel folds do not require novel Smotifs, but rather are a new combination of existing ones. Novel folds can be typified by the inclusion of a relatively higher number of rarely occurring Smotifs in their structures and, to a lesser extent, by a novel topological combination of commonly occurring Smotifs. When investigating the structural features of Smotifs, we found that the top 10% of most frequent ones have a higher fraction of internal contacts, while some of the most rare motifs are larger, and contain a longer loop region.

## Author Summary

Structural genomics efforts aim at exploring the repertoire of three-dimensional structures of protein molecules. While genome scale sequencing projects have already provided us with all the genes of many organisms, it is the three dimensional shape of gene encoded proteins that defines all the interactions among these components. Understanding the versatility and, ultimately, the role of all possible molecular shapes in the cell is a necessary step toward understanding how organisms function. In this work we explored the rules that identify certain shapes as novel compared to all already known structures. The findings of this work provide possible insights into the rules that can be used in future works to identify or design new molecular shapes or to relate folds with each other in a quantitative manner.

## Introduction

Under physiological conditions most proteins self-assemble into unique structures that dictate their interactions with other molecules and determine their function. Protein structures can be decomposed into individually folding units, so called folds [1]. A fold is determined from the number, arrangement, and connectivity (topology) of secondary structure elements [2]. Manually curated [3], semi–automated [4] and automated approaches [5], [6] classify protein folds by organizing them into hierarchical systems. Due to the lack of a clear understanding of how to define and classify folds, these various subjective approaches carry substantial inconsistencies [2], [7]. Meanwhile, recent studies paint a more nuanced picture of the fold universe of proteins, one that is more continuous in nature, where some higher density hubs formed by related structures correspond to and connect known folds [8], [9], [10], [11]. Part of the motivation to rethink the nature of the protein fold universe is provided by the apparent success of molecular modeling efforts that use short amino acid segments from known protein structures to build up novel folds [12]. Additional motivation comes from anecdotal examples that identify structures representing transitions between previously described folds, which either results in a unification of different fold families or suggests removing fold definitions altogether [13], [14]. One such example is described for the RIFT domain, where it is suggested that starting from an ancestral RIFT domain a strand invasion and a strand–swap event (with subsequent duplication and fusion events) resulted in the emergence of the swapped hairpin and double-psi beta barrel folds, respectively [15]. These folds cannot be interconverted with simple topological modifications, such as circular permutation, although their common evolutionary origin has been established.

Since the definition of complete folds is ambiguous, one has to consider structural definitions of smaller (local) entities, such as supersecondary structure elements, that could describe protein folds and the structure universe in a more quantitative and systematic nature. Supersecondary structure elements are defined as a number of regular secondary structure elements that are linked by loops (e.g. Rossmann, helix-turn-helix, four strand Greek key, β-meander motifs etc.). Folds are formed by the overlapping combination of various supersecondary elements, which are shared among different proteins and sometimes highly repeated within the same one. This observation prompted the theory of a relic peptide world [16], which proposes that modern, stable proteins are the results of duplication, mutation, shuffling and fusion of a limited set of relic peptides. Various efforts have tried to explore possible tool sets of supersecondary elements, such as antiparallel ββ-sheets [17], αββ and ββα motifs [18], αα-turn motifs [19], four helix bundles [20] and so on. Building on these earlier efforts, we introduced a new, general, supersecondary structure classification that fully describes all known protein structures [21]. In this schema a basic supersecondary motif, which we will refer to as Smotif, is composed of two regular secondary structure elements linked by a loop. Smotifs are characterized in protein structures by the types of sequential secondary structures and the geometry of the orientation of the secondary structures with respect to each other, as described by four internal coordinates [21], [22]. The definition for supersecondary structure elements for Smotifs is different from other studies or from the above mentioned textbook examples and it is rooted in practical reasoning. In this study we explored Smotifs of only two connected secondary structures because for this subset we had indication from prior work that the number of possible combinations are limited. Also, if we used a definition that has higher number of connected secondary structures e.g. 3 or more, the number of combinations would be very large and would prevent us from a systematic classification. Recently, we demonstrated that Smotifs with loop fragments having lengths up to 12 residues, together with their bracing secondary structure elements are exhaustively sampled in the Protein Data Bank (PDB). We also demonstrated that the available set of Smotifs has been essentially unchanged at least for the last 5 years, despite that during this time the sequence databases have doubled and a significant number of new folds have emerged [23].

These previous observations motivated us to analyze the occurrence of Smotifs among protein folds and explore the question of what is really unique about a structure that is identified as “novel”. Does the emergence of a novel fold coincide with the emergence of novel Smotifs that are integrated into a structure with known ones? Is it possible to generate novel folds solely from existing Smotifs? What are the rules that guide combinations of Smotifs to an apparently novel fold? Is the novelty of a certain Smotif or the novelty of combining well-known Smotifs the driving force behind the appearance of novel folds? These questions might be relevant to shed light on the rules governing protein structure evolution. There are practical considerations to understanding the actual limits of the definition and novelty of a fold. Exploring these issues can aid in developing more accurate structure modeling tools and support the design and realization of new and experimentally accessible molecular shapes.

## Results

### Smotif geometrical classification and saturation in the PDB

**Figure 1:**
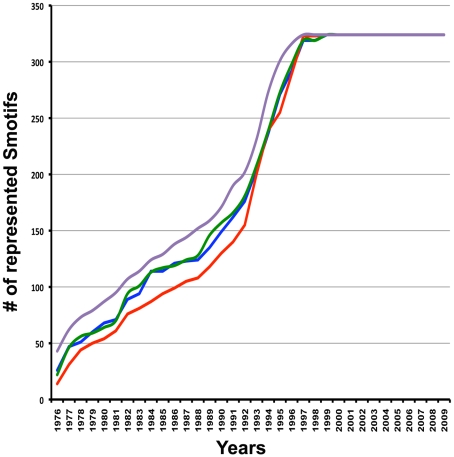
The saturation of Smotifs over time. Each curve corresponds to one of the four Smotifs categories (purple (strand-strand), green (strand-helix), blue (helix-helix) and red (helix-strand)). The cumulative distribution on the plot is obtained by summing the first appearances of Smotifs in 324 geometrical definitions as a function of time.

We explored the frequency of occurrences of all Smotifs in all protein folds. We established an exhaustive library of 324 types of Smotifs, as classified by their geometry, for each of the four combinations of possible bracing secondary structure elements. We have shown that this geometrical classification of Smotifs correctly captures local structural similarity (see Definition of optimal classification of Smotif geometry in Material and Methods). Previously we have shown that Smotifs are useful for loop prediction because loop conformations (as defined by the orientation of the embracing secondary structures) up to 10–12 residues are exhaustively sampled in PDB [21], [23]. We further refined this observation by exploring the increase of coverage of Smotifs in PDB over time (Fig. 1). Approximately 10 years ago all categories of Smotifs were already represented by at least one example.

### Structural factors affecting Smotif occurrence

**Figure 2:**
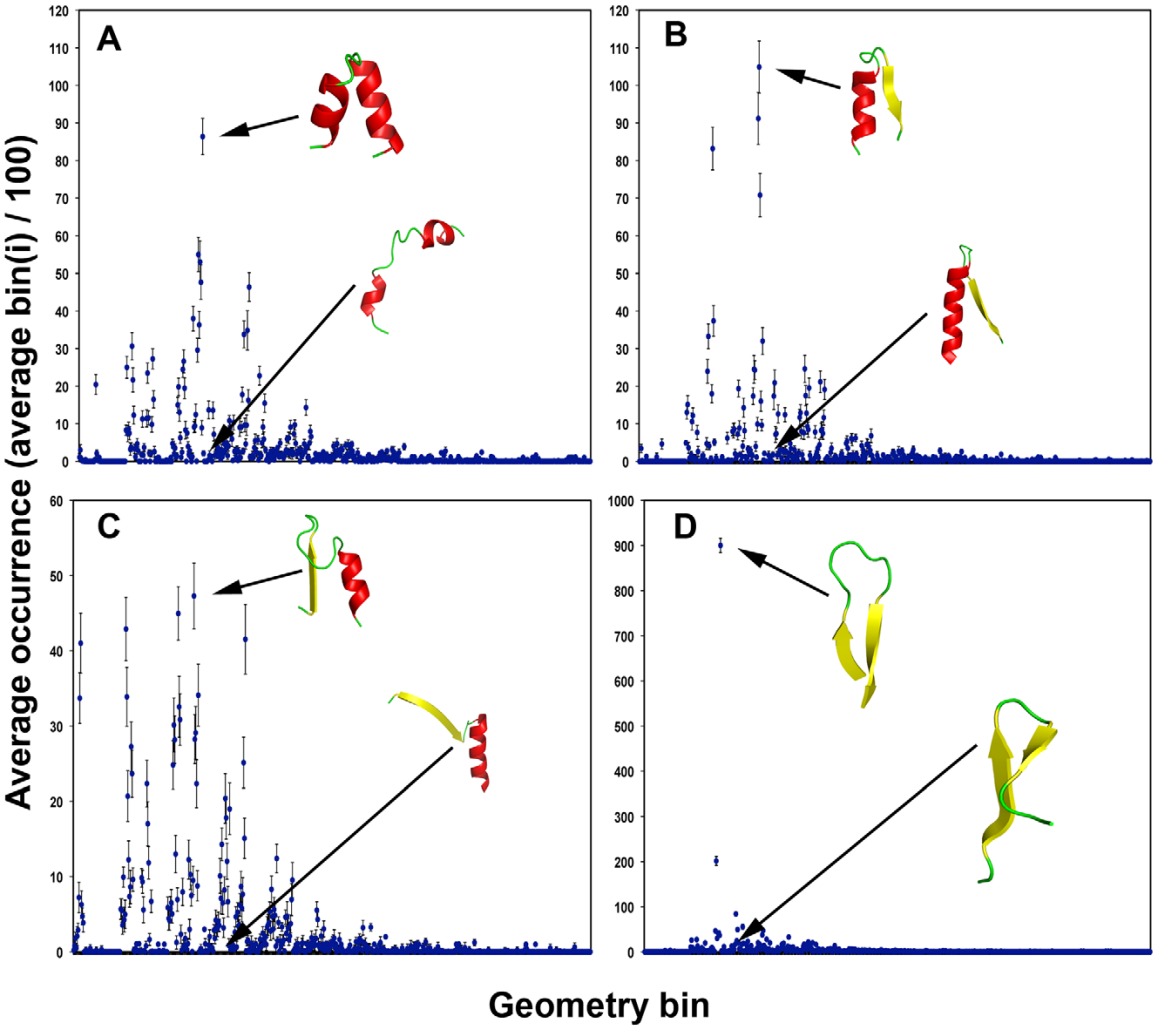
Average occurrence of Smotifs in all possible geometrical bins used for classification. Smotif frequencies are shown separately for types of α-α (A), α–β (B), β–α (C), and β–β (D). A non-redundant library of folds (one randomly picked structure from each SCOP fold class) as decomposed in Smotifs and the distributions are shown. Standard deviations are shown as extension bars and were obtained by repeating the random selection process 100 times.

**Figure 3:**
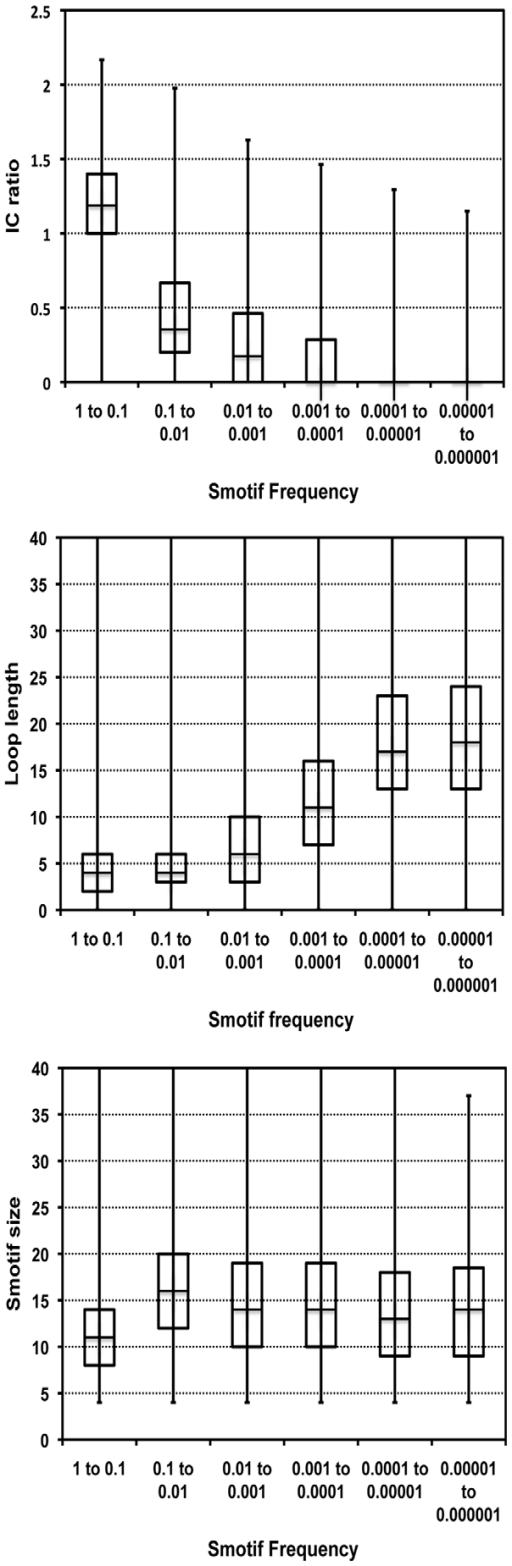
Box plots of various structural features of Smotifs as a function of Smotif frequency. IC ratio refers to the average number of internal contacts per residue; loop length is the length of the connecting segment between the two regular secondary structures within Smotifs, and Smotif size is the total number of residues in the Smotifs.

The occurrence of Smotif geometries in different types of protein folds is uneven (Fig. 2). There are some Smotifs whose geometries are ubiquitous, and occur in many different folds, while others are specific to a few. Fig. 2 displays a ββ class Smotif *(a)* that is highly represented across different folds, corresponding to a geometry that tightly aligns two ββ-strands and, thus, allows many non-bonded contacts to be formed. Meanwhile another Smotif within the ββ class (*b*), which is structurally similar but where one of the β-strands is tilted, has a very low occurrence within known folds. Similar trends can be observed for αα, αβ, and βα Smotifs: Smotifs forming extensive non-bonded interactions occur more frequently in known folds. We explored the normalized number of intra-motif non-bonded contacts as a function of Smotif frequency and found an exponential correlation between the number of contacts and frequency of motif usage (correlation of r = 0.83 as fitted on a logarithmic scale), indicating that the most frequent motifs (top 10%) are forming more contacts. However, there is not a statistically significant correlation for the rest of the Smotif frequencies (Fig. 3).

Another suspected factor for Smotif preferences is their size, as large Smotifs simply cannot fit into smaller folds. Here we found no clear tendency except once again the top 10% most frequent Smotifs, which indeed tend to be smaller (on average 12 (σ = 6) residues total within the bracing secondary structures, without counting the variable number of loop residues, while motifs at all other frequencies are generally formed by 16 residues (σ = 8)). The longer the loop connecting the bracing secondary structures, the more likely that contacts will be formed between non-proximal secondary structures: e.g. a ββ-type Smotif that connects together strands of two β-sheets. A correlation was found between the length of the loop within Smotifs and the frequency of Smotif usage in folds among the 50% least frequent Smotifs. However, Smotifs extracted from new folds do not show correlation between Smotifs size or loops length and the frequency of Smotifs: i.e. new folds are not necessarily formed by large Smotifs and do not necessarily have particularly long loops (data not shown).

We also explored whether solvent accessibility is correlated with the frequency of Smotifs, as one could suspect that buried, conserved cores would be formed by frequently occurring Smotifs and structural regions outside the common core would have a trend to comprise a higher proportion of rare Smotifs, due to a less restrictive structural environment. However, we could not find any statistically significant correlation between the frequency of Smotifs and their exposure (Fig. S1).

### Smotif distribution in novel and known folds

**Figure 4:**
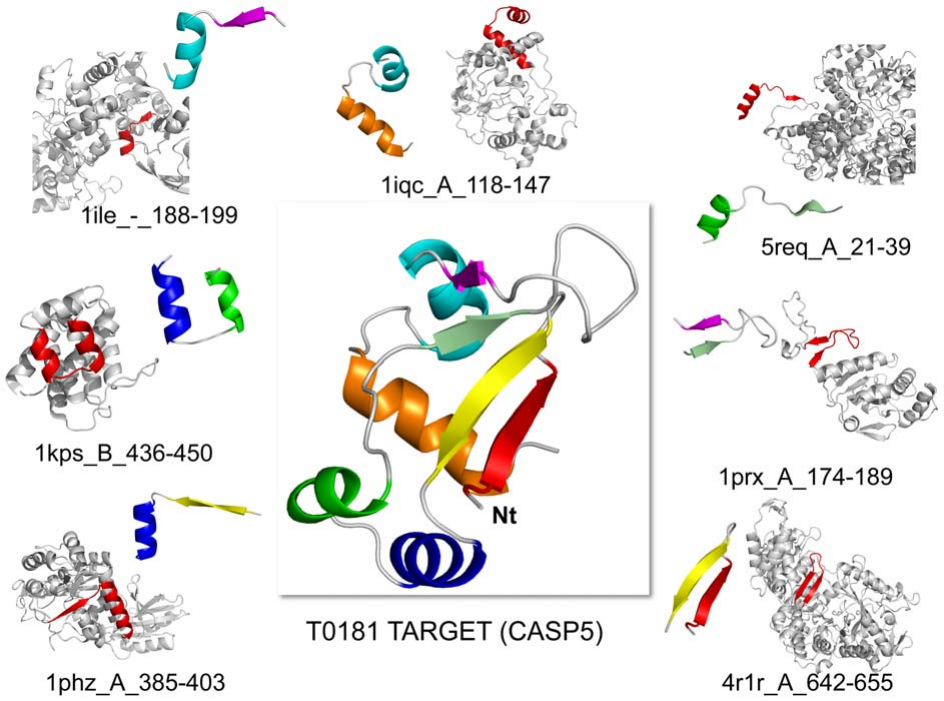
Decomposition of target T0181 from CASP5 into Smotifs and matching of Smotifs to existing protein structures. Each of the Smotifs was used as a probe to search a backdated database of protein structures. The PDB code, chain, start, and end residue position that match the specific Smotif is shown below each structure.

Since the repertoire of Smotifs seems to have come close to saturation (Fig. 1) [23], this prompts the question of what is really unique about a fold structure when it is identified as “novel”. Detecting novel folds is a non-trivial question. Automated structural comparisons are often followed by manual inspection to characterize new protein structures. We have explored proteins that were classified as novel at the time of their discovery in two expert validated sources, in the archives of SCOP [3] and in the series of CASP experiments [24]. We found that proteins that were considered novel folds at CASP 3–6 meetings (years 1998–2004) and in SCOP 1.73, 1.75 (years 2007–2009) do not have any novel Smotif geometries that were not present in previously solved structures. In other words, none of the Smotifs of novel folds have a unique geometry (Table 1). For instance, as early as the third round of CASP Meetings in 1998 [25], all of the targets identified as novel folds by the experts could have been reconstructed using Smotifs from known protein structures. If, in our Smotif comparison, we required not only a match in the geometry between the Smotifs in the novel structures and those in the solved structures, but also required identical lengths of the flanking secondary structures, still less than 6% of the Smotifs in novel folds at CASP meetings would not have a match in already known structures. Similarly, we have checked the motif composition of new folds from the archives of SCOP in the 1.73 (2007 November) and 1.75 (2009 June) releases. These contain a total of 233 new folds from 1140 proteins. Similar to the CASP targets, none of these novel folds had a Smotif that was not already observed in a previously known fold. With the stricter definition, that requires a fit of the length of the bracing secondary structures, still less than 1% proved to be novel Smotifs. Initially, we found 47 Smotifs (out of the 8056 analyzed) that appeared to be new. However, after manual inspection, it turned out that these are all explained by an artifact of replacing obsolete PDB entries with newer ones, with a corresponding newer date. The above observations suggest that recently solved novel folds do not imply the emergence of new Smotifs, and that a protein with a novel fold can be constructed using Smotifs from already existing protein folds. As an illustration, T0181 (PDB code: 1nyn), a new fold submitted to CASP5, can be constructed from 7 overlapping Smotifs, all of which can be located in previously solved structures of other proteins representing a variety of different folds (Fig. 4).

**Table 1:** Summary of geometry and SSs among CASP targets and SCOP folds considered as ‘New folds’.

CASP meeting	# Smotifs	# Smotifs with new geometry^a^	# Smotifs unique FSS^b^
3	62	0	2
4	72	0	3
5	42	0	4
6	59	0	4
SCOP dataset			
1.73	4567	0	45
1.75	3489	0	42

a Number of Smotifs with new geometrical classification after comparing with Smotifs extracted from protein structures already known.

b Number of Smotifs that are formed by flanked secondary structures (FSS) of SS1, SS2 with unique lengths as compared to all previously known. For example, protein 1fw9 chain A was considered a new fold during CASP4 meeting (target id. T0086). It has a ββ motif between residues 73 and 95. The specific Smotif geometry was present in the backdated protein databank, but none of the Smotifs with the same geometry had two beta strands with comparable length (beta strand lengths are 10 and 11 residues for SS1 and SS2 respectively).

**Figure 5:**
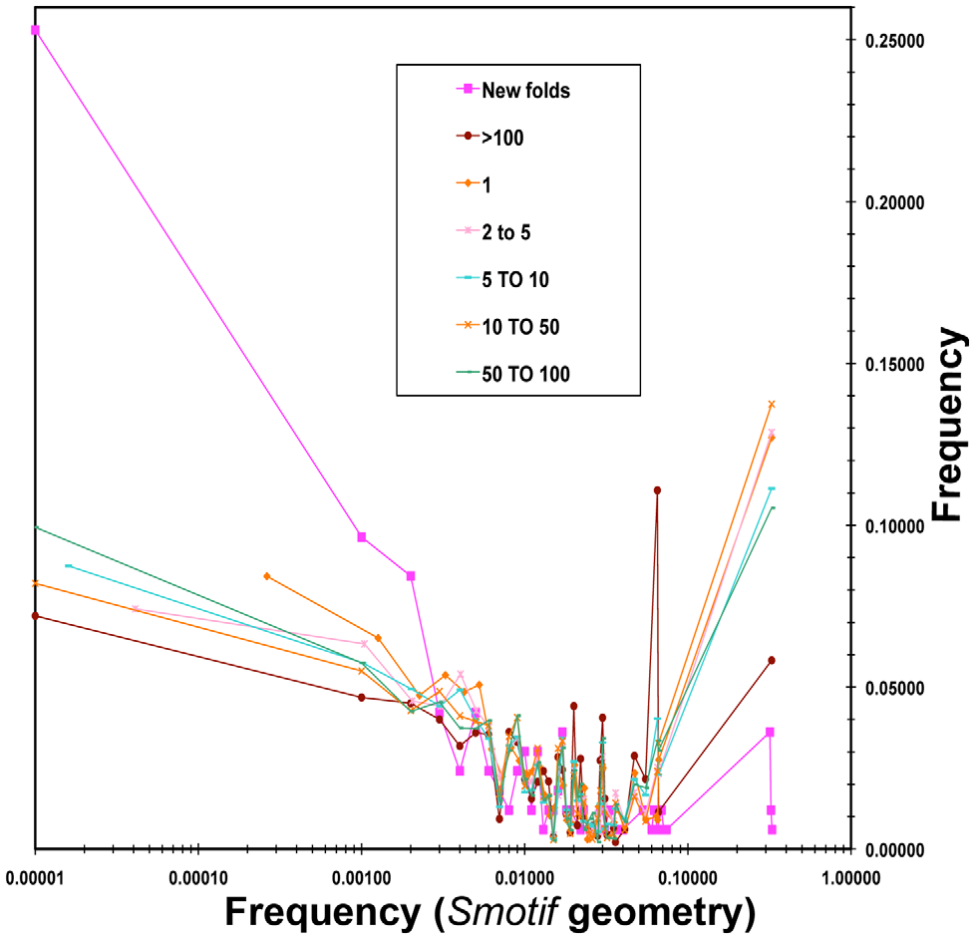
Distribution of the frequency of Smotif geometries in CASP datasets. Proteins were grouped according to the number of structures per fold. Seven categories were described: new folds (blue romboid); folds with: one protein (green triangle), 2 to 10 (purple box), 10 to 50 (cyan box), 50 to 100 (orange circle), and more than one hundred proteins (red box), respectively. The values were plotted as histogram of frequencies with a log scale in the X-axis. The same dataset and approach is used to avoid redundancy as for Fig. 2.

When we explored the frequency of occurrence of Smotifs in the non-redundant set of known folds, we observed that novel folds have a larger fraction of Smotifs that have a low frequency of occurrence in the PDB (Fig. 5 CASP dataset; see Fig. S3 and S4 for distribution of Smotif frequency calculated for SCOP 1.75 and SCOP 1.73 respectively). On the other hand, superfolds [26], those that are adopted by many different sequences often with different functions, are built by Smotifs that occur with medium or high frequencies in existing folds. This implies that novel folds are composed of a new permutation of existing Smotifs and, specifically, a structure will have a greater likelihood of being “novel” if the structure is enriched with rarely occurring Smotifs. This phenomenon becomes especially apparent when the relative frequency of occurrences of Smotifs drops below 0.09 (Fig. 5, Fig. S2, Fig. S3).

**Figure 6:**
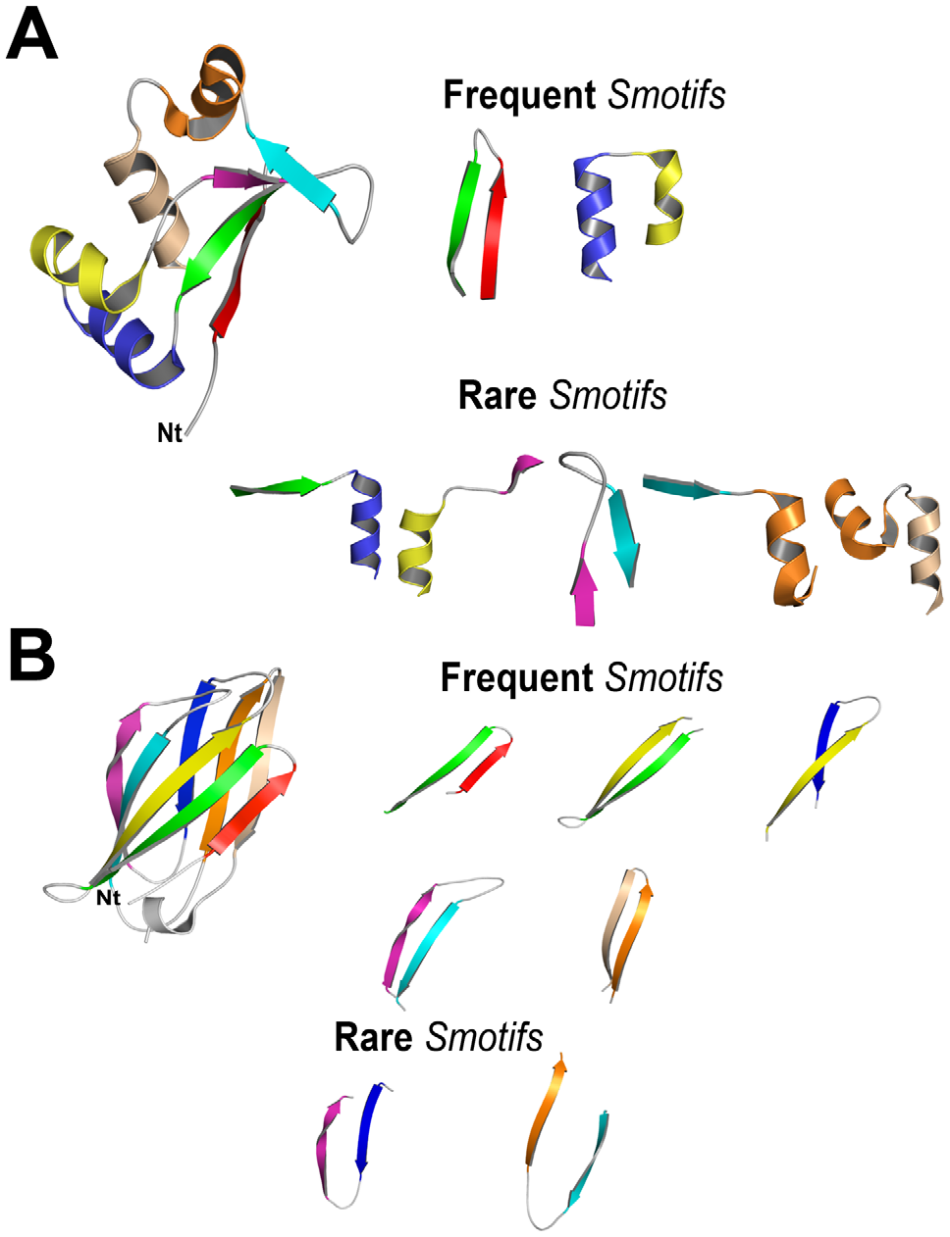
**A.** Example of a novel fold protein, target T0181, submitted to CASP5 meeting. The protein contains two Smotifs with a geometry considered as high frequent and five Smotifs with rare geometries. The PDB code is 1nyn. **B.** Example of a protein from a highly populated fold, Immunoglobulin-like beta-sandwich (SCOP fold id. 48725). Five out of the seven Smotifs that form the protein structure have geometries with high frequencies. The PDB identification code is 1byv.

Two examples of the above observations are illustrated in Fig. 6. The first example is the new fold target T0181, discussed above (PDB code: 1nyn; Fig. 6A). The second example is a member of the immunoglobulin fold (PDB code: 1gyv; Fig. 6B), which is one of the most populated folds. Target 181, a new fold structure, can be decomposed into 7 Smotifs, where five are considered low frequency (i.e. frequency smaller than 0.01, or less than 1%). On the other hand, for a representative structure of the immunoglobulin fold (SCOP fold descriptor 48725, Immunoglobulin-like beta sandwich), the opposite situation occurs. Five out of the 7 Smotifs that comprise the structure are very well represented (high frequency) in the pool of Smotifs (Fig. 6B).

**Figure 7:**
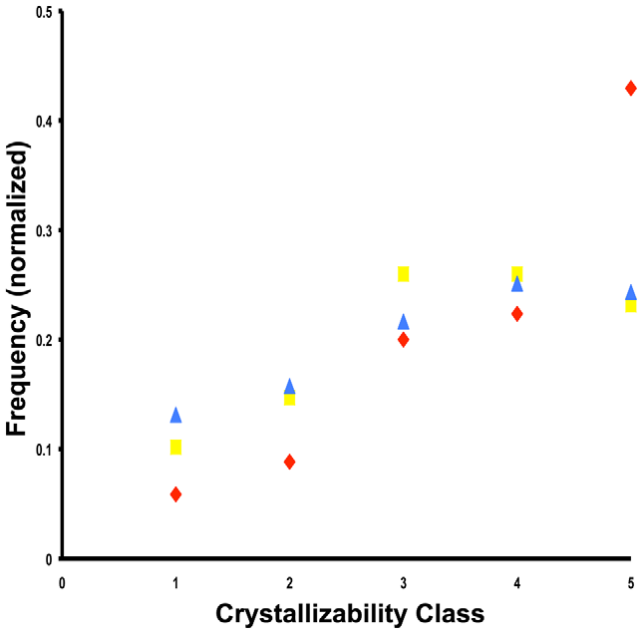
XtalPred crystallizability score distribution for new and known folds. The normalized frequencies of crystallizability class scores (1 = optimal to 5 = very difficult) are plotted for domains from new folds in SCOP 1.75 (red diamonds) and in SCOP 1.73 (yellow squares), respectively, and for known folds (blue triangles).

One could speculate that some novel folds were recently discovered simply because of difficulty in experimental determination, i.e. these structures are harder to solve. We used the XtalPred program [27] to predict the crystallizability of 347 new folds and 2802 known folds, all solved approximately in the same time period (since SCOP 1.73 released in 2007). We found that new folds from the most recent SCOP release 1.75 indeed have a small tendency to be less feasible for experiments. However, XtalPred and other prediction methods for protein crystallizability heavily rely on known homologs of a query sequence. The rationale is that if a protein with a similar sequence has been solved before it usually indicates that this particular protein family is more experimentally tractable. This artifact is illustrated in our analysis by the fact that while new folds from SCOP 1.75 do show less favorable XtalPred scores as compared to known folds, this difference disappears in case of new folds of SCOP 1.73 (Fig. 7).

### Novel folds as an unusual combination of common Smotifs

**Figure 8:**
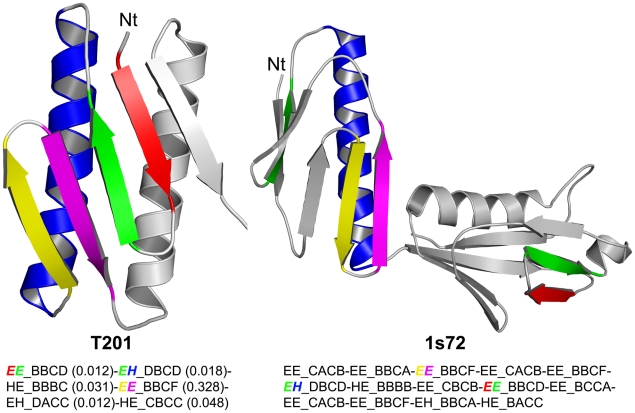
Example of a new protein fold, target T0201 that does not contain rare Smotifs. Three out of the six Smotifs that compose target T0201 are also present in the 50S ribosomal protein L6P (PDB code 1s72 chain E) but in a different topological arrangement. Structurally equivalent Smotifs between T201 and 1s72 are depicted in the same color-coding. The sequence of Smotifs is also shown underneath for each protein. In each Smotifs description the first two letters refer to the two secondary structures connected (E and H stand for strand and helix, respectively). The 4 letters after the underscore sign code the 4 geometrical variables describing the relative geometry of the Smotif (in order: the distance between the bracing secondary structures, and three angles: a hoist (δ), a packing (θ) and a meridian (ρ) [21]). For T201, the relative frequency of motifs is also indicated in brackets. Within the Smotif descriptions, the color-coding helps to locate the corresponding secondary structures in the ribbon models. Equivalent Smotif descriptions between the two proteins (in general all of them are shown in italics) refer to geometrically equivalent Smotifs.

Another plausible way to generate new folds is to combine, otherwise common Smotifs in an unusual sequence, to result in a new topology. To explore this, we calculated a Novelty Z-score for each protein, which was obtained from the product of individual Smotif frequencies. The hypothesis is that if the Novelty Z-score of some novel folds is similar to that of known folds, then the novelty for these cases must be a consequence of a never before seen combination of otherwise common Smotifs rather than a result of being constructed from rare Smotifs. And while new folds from the CASP dataset do show a distribution of Novelty Z-scores biased towards low values (Fig. S4), in the case of SCOP 1.75 (Fig. S5) and SCOP 1.73 (Fig. S6), most novel folds are indistinguishable from already known structures in terms of their overall Novelty Z-scores, which indicates that these structures may indeed be a new topological arrangement of common Smotifs. However, one may note the more frequent extreme negative outliers in the distributions for the novel folds in these datasets (averages and standard deviations are −1.03±1.1, 0.25±1.35 and 0.0±1.0 for CASP dataset, SCOP 1.75, and SCOP 1.73, respectively). This means that although novel folds are often built using a higher proportion of rare Smotifs, in many cases these folds are novel because their Smotifs are assembled in an unusual sequence. This is illustrated with Target T0201 (CASP 6) and the S50S ribosomal protein L6P (PDB code 1s72 chain E) that share 3 out of 6 of their Smotifs (Fig. 8). However the sequential arrangement of these shared Smotifs is different, yielding different topologies.

## Discussion

Since the early nineteen-nineties, it has been clear that the universe of protein folds is much more limited and redundant than the sequences adopting them [28]. Structural biology and the recently launched Structural Genomics efforts have discovered a large subset of possible fold shapes. Many predictions suggest that most of the folds are already known [28], [29], [30]. Meanwhile, by solving many of the possible folds, the characteristic differences earlier described among fold definitions has become more blurred [8], [10], [31]. In practice, discovering all possible folds may be an impossible task, partly because it is clear now that the definition of folds is highly subjective [2], and partly because the distribution of folds is extremely uneven: while only a dozen superfolds seem to populate half of a typical genome, and only about 200 folds populate 2/3 of it, it is possible that many thousands of more rarely occurring shapes need to be discovered to reach 80–90% coverage of all possible shapes that were established during evolution [32] [33].

In this work we explored the entirety of protein shapes from the perspective of their Smotif building blocks, which can be defined more objectively than the folds themselves, and which are observed to be nearly completely sampled in the currently known structures. Using this repertoire of Smotifs, we observed that novel folds can be distinguished from already discovered ones by the presence of rare Smotifs and, less often as an unusual combination of otherwise common Smotifs. The most frequently used motifs have a higher average number of internal contacts, while some of the rarest motifs are larger, and contain longer linker regions. These observations may be useful starting points for future works to identifying or designing sequences that are likely to constitute “novel” folds.

While in this work we defined Smotifs according to practical considerations and did not investigate if these Smotifs or subset of them could also serve as possible units for structural evolution, it is noteworthy to mention other studies that identified similar structural elements as possible building blocks of structural hierarchy using different approaches. The so called *Closed Loops* were identified by their close Cα-Cα contacts from solution structures and found to have a nearly standard size (27 residues +/−5). This typical size distribution of Closed Loops was supported by polymer statistics, as it is the theoretical optimal size for loop closure and subsequently suggested to be a universal building block of protein folds [34], [35]. In another approach, dynamic Monte Carlo simulation of alpha carbon chain of the nearest 24 neighbor in a lattice model identified clusters of “most interacting residues”, which serve as anchors for protein folding [36]. These anchors were found to be conserved hydrophobic clusters of residues that keep together the so called *Tightened End Fragments*, which essentially correspond to the Closed Loop definition. Finally a most recent paper updates on the idea of ancient relic peptides of length 20–40 residues that co-occur in different structural contexts, and suggested to be an ancestral pool of peptide modules [37].

## Materials and Methods

### Protein structure datasets

All structures from CASP 3,4,5,6 meetings [38] that were manually identified as “novel folds” at the time of the experiment: CASP3 (protein identification (PDB code): T0052 (2ezm), T0059 (1d3b), T0063 (1bkb), T0067 (1bd9), T0071 (1b9k), T0080 (1bnk), and T0083 (1dw9)), CASP 4 (T0086 (1fw9), T0116_3a (1ewq), T0116_3b (1ewq), T0120_1 (1fu1), and T0124 (1jad)), CASP5 (T0129 (1izm), T0149_2 (1nij), T0161 (1mw5), and T0162_2 (1izn)) and CASP6 (T0201 (1s12), T0209_2 (1xqb), T0216_1 (1vl4), T0216_2 (1vl4), T0238 (1w33), T0242 (2blk) and T0248_2 (1td6)) were collected. Four tailored datasets of previously solved protein structures were generated for comparisons with the “novel” folds of each CASP experiment (see below). The tailored datasets did not contain any structure that was deposited after June 1998 (6,366 entries), June 2000 (10,199 entries), June 2002 (15,234 entries) and June 2004 (22,076 entries) to compare with targets from CASP3, CASP4, CASP5, and CASP6 respectively. Similarly, four SCOP [3] database releases were used for calculating motif frequencies (see below): SCOP 1.39 (CASP3 new fold set), SCOP 1.53 (CASP4 new fold set), SCOP 1.61 (CASP5 new fold set), and SCOP 1.69 (for CASP6 new fold set). Since CASP meetings start in June and SCOP databases were released after June during the same year, all structures that were present in the SCOP database with a deposition date after June were removed.

Similarly, we have downloaded all “new folds” from the SCOP 1.73 and 1.75 releases, 123 and 110 folds, respectively, that are part of a total of 1140 proteins. The list of new folds for earlier releases can be found at SCOP via History link (http://scop.mrc-lmb.cam.ac.uk/scop/index_prevrel.html).

### Definition of an optimal classification of Smotif geometry

A Smotif is defined by two consecutive regular secondary elements (i.e. α-helix or β-strand), connected by a loop. The N and C-terminal regular secondary structures of a Smotif are referred as SS1 and SS2, respectively. Motif geometry refers to the local spatial arrangement of SS1 with respect to SS2 as introduced in [22] using four internal coordinates. Briefly, SS1 and SS2 were represented by their principal moments of inertia (M1 and M2). If P1 and P2 are the end point of SS1 and start point of SS2, and L is the vector between P1 and P2, then plane Π is defined by M1 and L and plane Γ is defned by M1 and the normal to plane Π. Geometry of a Smotif is expressed by four measures: the distance (D) between the C-terminal of SS1 and the N-terminal of SS2 (distance between P1 and P2) and three angles: a hoist (δ): angle between L and M1, a packing (θ): angle between M1 and M2, and a meridian (ρ): angle between M2 and plane Γ (Fig. 2 in [21]).

A library has been established that classifies each Smotif in all PDB structures. This library is organized in a two-level hierarchy: in the first level of classification, (i) Smotifs are identified according to the type of bracing secondary structures: αα, αβ, βα and ββ according to the definition of secondary structure by the DSSP program [39]. At the second level, (ii) Smotifs are grouped according to their geometry, as described above [21], [22]. A protein structure can, therefore, be expressed as a string of overlapping Smotifs where the SS2 from one Smotif constitutes the SS1 in the following Smotif.

The geometrical values used in the second level of classification are distributed in a continuous space. Distance is distributed between 0 and 40 Å. (values larger than 40 Å are assigned to 40), δ and θ angles span from 0 to 180 degrees, and the ρ angle spans from 0 to 360 degrees. In order to compare Smotif geometries, the parameter spaces of geometrical values were binned, where each bin is defined by the 4 parameters described above. A range of binning sizes and parameter intervals were explored for the four variables in order to get the sharpest partitioning power of the geometrical space with the smallest number of possible bins (Fig. S7). The quality of the binning was assessed by calculating the RMSD (Root Mean Square Deviation) and the LGA scores [40] upon structural superposition for all Smotifs that were classified in the same or different geometrical bin. The optimal bin partitioning for each parameter was obtained by studying the distribution of distance and angle values of Smotifs in SCOP 1.71 proteins and resulted in only 324 types of Smotif definitions using the following binning values: 4 Å bins for distance, 60 degree bins for δ and θ starting at 0 degree, and 60 degree bins for ρ, starting at 30 degree. At this level of bin resolution the RMSD upon structural superposition of more than 75% of Smotifs that belong to the same geometrical bin falls below 1 Å (Fig. S7).

A program that defines Smotifs is available upon request from the authors.

### Comparing geometries of Smotifs in novel folds with previously known structures

All protein structures that were identified as “new folds” from SCOP releases 1.73 and 1.75 and CASP 3–6 meetings were decomposed into Smotifs. In case of SCOP, each release identifies the new folds in comparison to the rest of the folds while in case of the CASP sets a Smotif library extracted from a backdated PDB was prepared for each CASP meeting. Within the pairs of datasets, Smotifs in SCOP new and existing folds and Smotifs from CASP new folds and the corresponding Smotif library from previously solved structures, were compared to evaluate the existence of identical Smotifs in the novel folds and the previously defined folds. The first comparison was based on the type of secondary structures and the geometry (D, hoist, packing, and meridian) of Smotifs. In a second, stricter comparison, the lengths of the flanking secondary elements (SS1 and SS2) were also compared. If these lengths differed by more than 2 or 4 residues in the case of strands or helices, respectively, the Smotifs were considered different.

### Calculating frequencies of occurrences of Smotif classifications

To avoid redundancy when calculating the frequencies of Smotif occurrences for each four-dimensional geometric bin, only a single protein was selected from each protein fold (as defined by SCOP database). Since fold families contain more than one protein structure and structures that belong to the same fold may have a variable number of Smotifs this selection process was repeated 100 times, randomly selecting a different protein in each analysis. Therefore, the frequency of occurrence of a given geometrical bin is the average of counts computed from 100 rounds of analysis for each family.

### Calculating Zscores for Novelty

Each of the proteins in the database was converted into a string of Smotifs. Thus, a protein having 5 regular secondary structures would be expressed as a string of 4 overlapping Smotifs. For each protein, a normalized probability score of observing such a string of Smotifs was calculated:
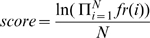
(1)where N is the number of Smotifs and *fr* is the frequency of the Smotif *i* as calculated previously. Individual scores were converted into statistical Z-scores using the mean (μ) and standard deviation (σ) of the population of scores, as (2)
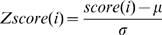
(2)


### Calculating non-bonded contacts in Smotifs

Internal contact ratio was calculated as the number of non-bonded atomic contacts (i.e. H-bonds, polar contacts, hydrophobic contacts) between SS1 and SS2 divided by Smotif size. Contacts were defined by the Contact of Structural Units (CSU) program [41]. CSU is based on the detailed analysis of interatomic contacts and interface complementarity. For every structural unit CSU calculates the solvent accessible surface of every atom and determines the contacting residues and type of interactions they undergo including all putative hydrogen bond contacts.

### Calculating crystallizibility

Protein crystallizability was predicted with the XtalPred server [27]. XtalPred predicts protein crystallizibility by combining nine features: length, length of predicted disorder, Gravy index, insertion score, instability index, percent of coil structure, isoelectric point. Based on these features the protein is assigned to one of five crystallization classes: optimal, suboptimal, average, difficult and very difficult. Each class represents different crystallization success rate observed in TargetDB [42]. Three SCOP domain datasets were compiled for submission to XtalPred; domains from “new folds” as defined in (1) SCOP 1.75 and (2) in SCOP 1.73, respectively, and (3) domains in SCOP 1.75 that were added since the release of SCOP 1.73 and that were not new folds. This ensures that we are focusing on proteins that were solved approximately in the same time but were classified differently in terms of novelty. The amino acid sequences of the domains were obtained from the ASTRAL website (astral-scopdom-seqres-gd-all-1.75.fa, astral-scopdom-seqres-gd-all-1.73.fa). Sequence redundancy was removed among the domains using CDHIT clustering [43] at 95% sequence identity threshold. The SCOP 1.75 and 1.73 “new fold” domains dataset contained 170 and 177 representative sequences (517 and 558 redundant sequences), respectively, and the SCOP 1.75 “known fold” dataset contained 2802 representative sequences (out of 13,043 redundant ones). Each amino acid sequence was submitted to XtalPred to calculate the crystallizability class.

### Solvent accessibility of Motifs

The corresponding PDB structure, chain identification and residue range was located for each Smotif (369,859 Smotifs in total). We calculated ACC values (water exposed surface area or number of water molecules in contact with the residue) using the DSSP program [44]. The average solvent accessibility of Smotifs was calculated by averaging the ACC values over all residues of the Smotif. We also calculated average ACC values by excluding loop residues, which are usually exposed, for each Smotif, but the conclusions were not affected.

## Supporting Information

Figure - S1 Solvent accessibility scores of Smotifs as calculated by DSSP. Average solvent accessibility values are plotted as a function of Smotif frequency in α-α (A), β-α (B), α-β (C), and β-β (D) Smotifs.
(5.67 MB TIF)

Figure - S2 Distribution of the frequency of Smotif geometries in SCOP 1.75. Proteins were grouped according to the number of structures per fold. Seven categories were described: new fold (blue rhomboid); folds with: 1 protein (green triangle), 2 to 10 (purple box), 10 to 50 (cyan box), 50 to 100 (orange circle), and more than hundred proteins (red box), respectively. The values were plotted as histogram of frequencies with a log scale in the X-axis. The same dataset and approach is used to avoid redundancy as in Fig. 2.
(6.42 MB TIF)

Figure - S3 Distribution of the frequency of Smotif geometries in SCOP 1.73. Proteins were grouped according to the number of structures per fold. Seven categories were described: new fold (blue rhomboid); folds with: 1 protein (green triangle), 2 to 10 (purple box), 10 to 50 (cyan box), 50 to 100 (orange circle), and more than one hundred proteins (red box), respectively. The values were plotted as histogram of frequencies with a log scale in the X-axis. The same dataset and approach is used to avoid redundancy as in Fig. 2.
(6.42 MB TIF)

Figure - S4 Histogram of Novelty Z-scores of known folds in CASP dataset. Z-scores were binned by increments of 0.1. Overlaid are the Novelty Z-score for each individual new fold target submitted to CASP meetings
(2.92 MB TIF)

Figure - S5 Histogram of Novelty Z-scores of known (red) and new (blue) folds in SCOP 1.75 dataset. Z-scores were binned by increments of 0.1.
(2.92 MB TIF)

Figure - S6 Histogram of Novelty Z-scores of known (red) and new (blue) folds in SCOP 1.73 dataset. Z-scores were binned by increments of 0.1.
(2.92 MB TIF)

Figure - S7 Structural similarity vs. geometry binning. Panels A and B show the distribution of LGA score [40] and RMSD (Cα) of pairs of Smotifs that share the same geometry bin for different of bin definition: red rhomboid: 2_45_45_45, blue circle: tailored binning (see Materials and Methods section), green square: 4_90_90_90, orange triangle: 8_90_90_180; where for instance the binning 2_45_45_45 means that D in binned in interval of 2Å, and δ, θ, and ρ angles in 45 degrees respectively. Panels C and D are analogous to A and B but result from the comparison of pairs of Smotifs that have different geometries.
(5.67 MB TIF)
