# Supplementary figures and images for: Structural Characteristics of Novel Protein Folds
Source: PLoS Comput Biol. 2010 Apr 22;6(4):e1000750. doi: 10.1371/journal.pcbi.1000750 (PMC2858679; doi:10.1371/journal.pcbi.1000750)

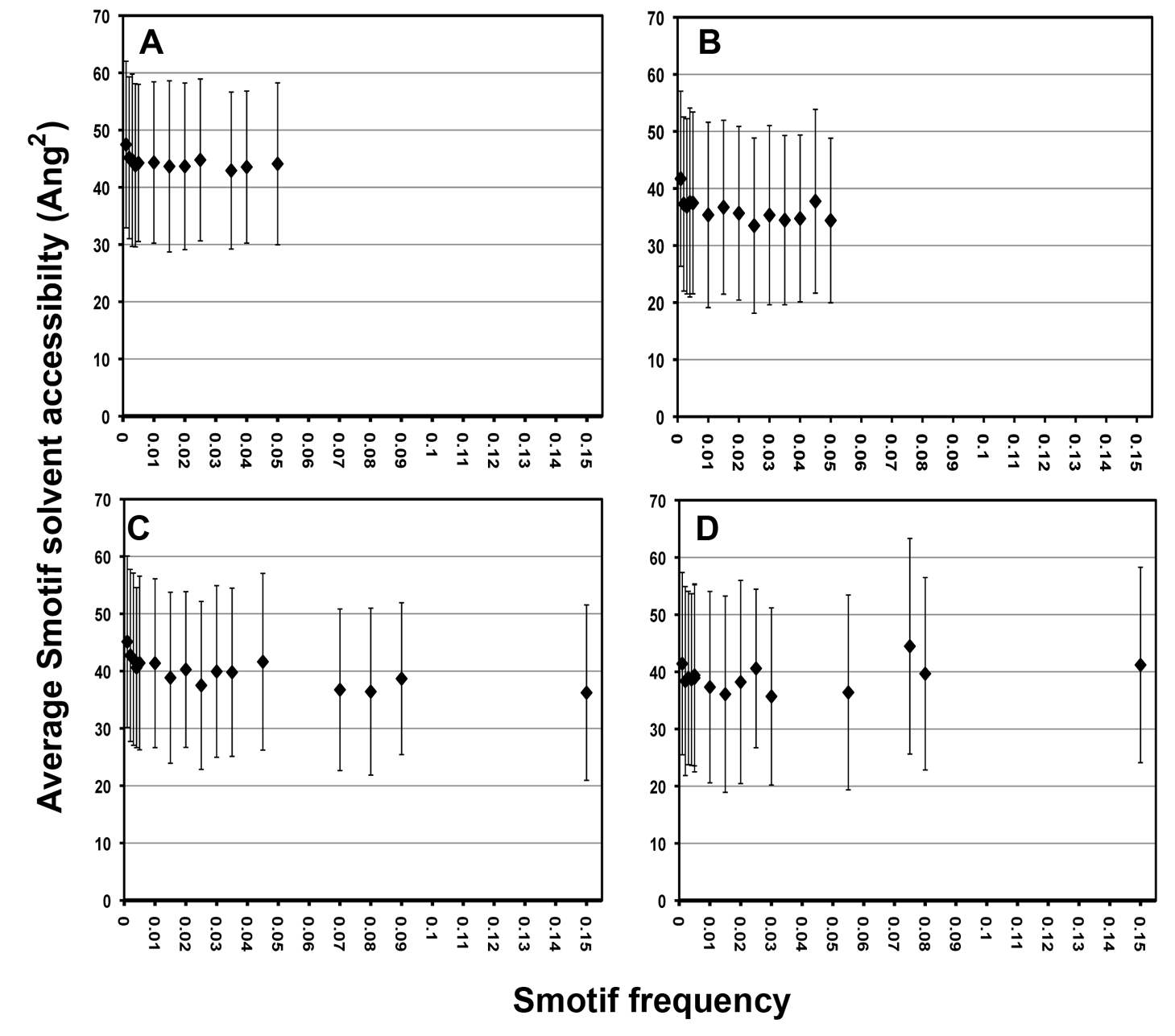

Supplement: Figure - S1 — Solvent accessibility scores of Smotifs as calculated by DSSP. Average solvent accessibility values are plotted as a function of Smotif frequency in α-α (A), β-α (B), α-β (C), and β-β (D) Smotifs. (5.67 MB TIF) [file pcbi.1000750.s001.tif]

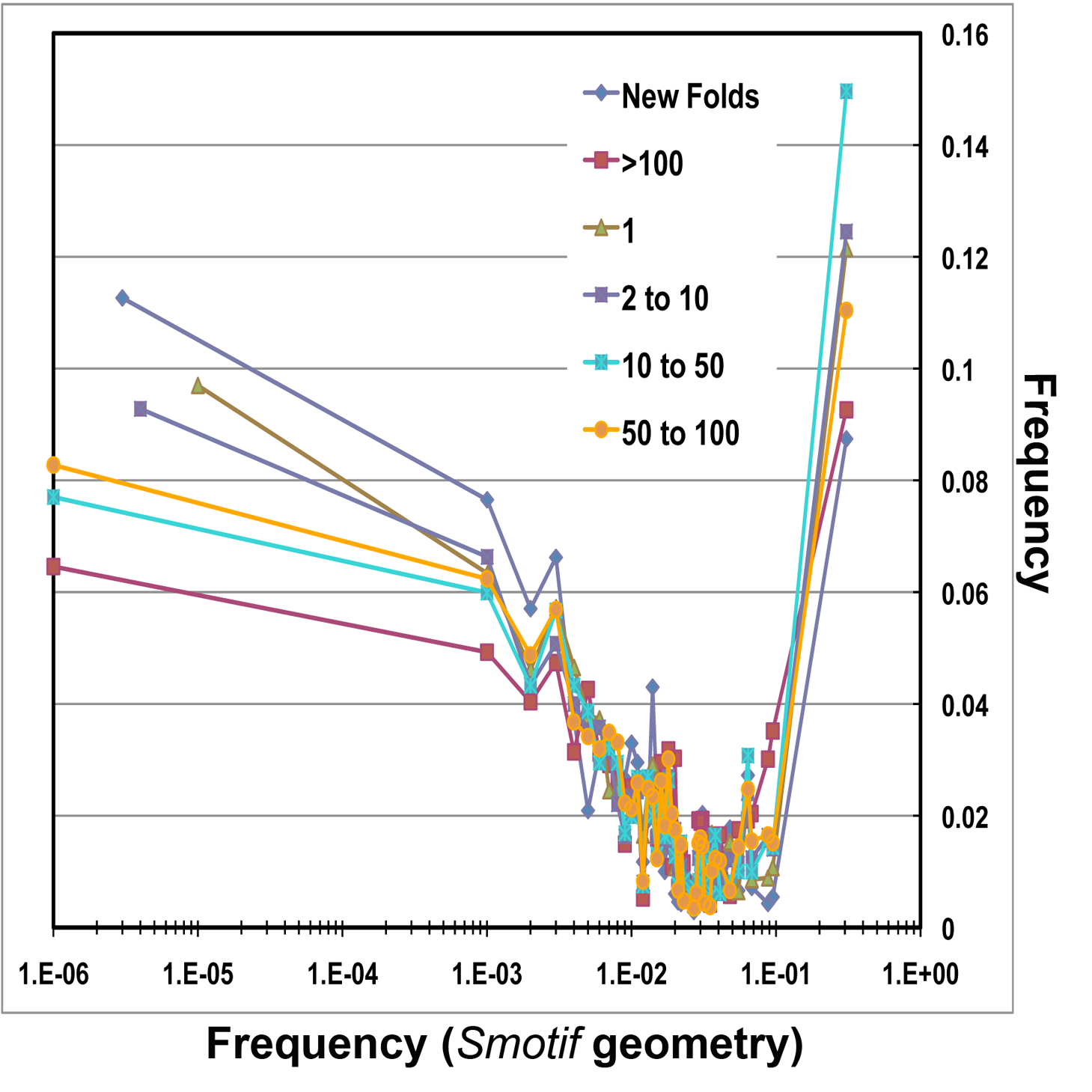

Supplement: Figure - S2 — Distribution of the frequency of Smotif geometries in SCOP 1.75. Proteins were grouped according to the number of structures per fold. Seven categories were described: new fold (blue rhomboid); folds with: 1 protein (green triangle), 2 to 10 (purple box), 10 to 50 (cyan box), 50 to 100 (orange circle), and more than hundred proteins (red box), respectively. The values were plotted as histogram of frequencies with a log scale in the X-axis. The same dataset and approach is used to avoid redundancy as in Fig. 2. (6.42 MB TIF) [file pcbi.1000750.s002.tif]

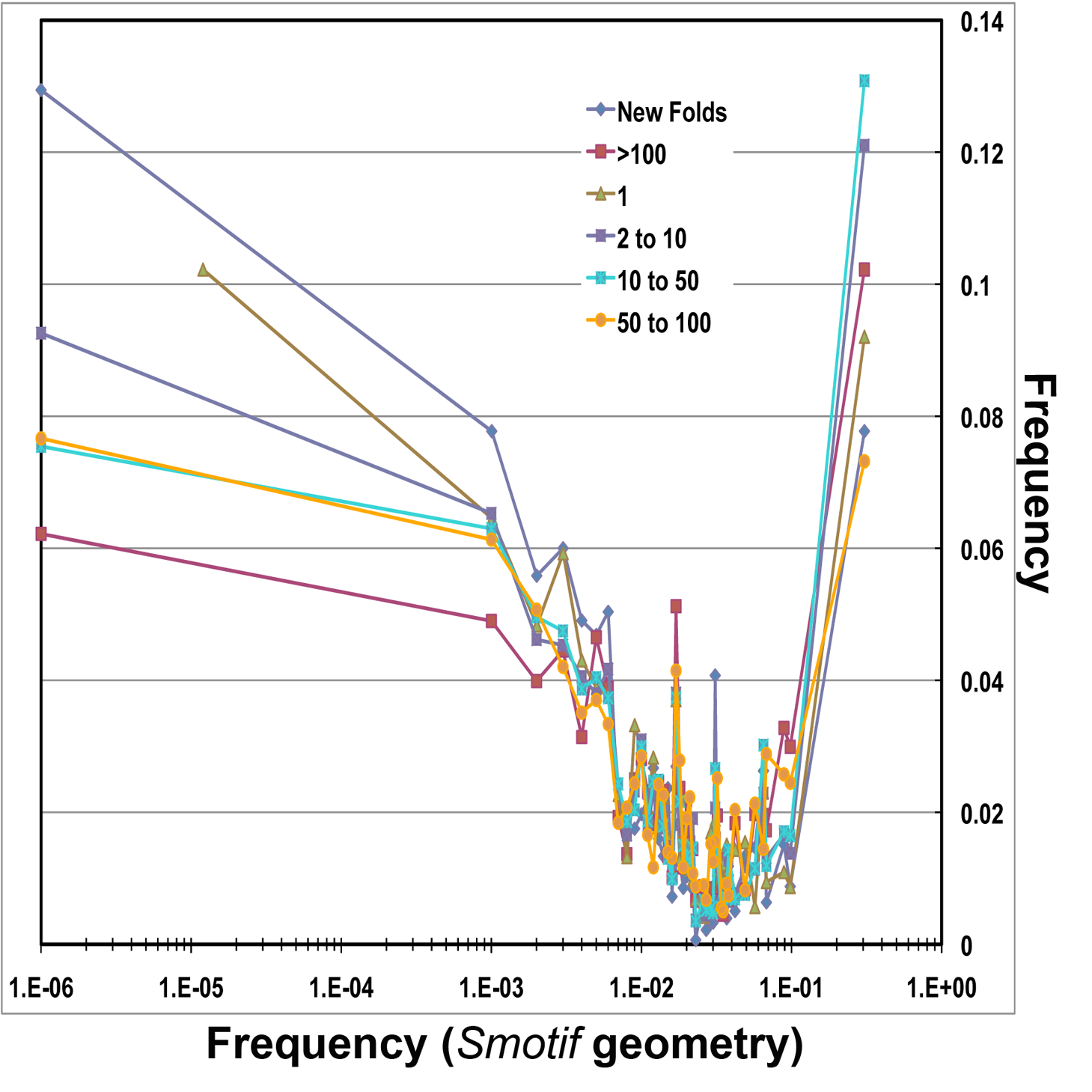

Supplement: Figure - S3 — Distribution of the frequency of Smotif geometries in SCOP 1.73. Proteins were grouped according to the number of structures per fold. Seven categories were described: new fold (blue rhomboid); folds with: 1 protein (green triangle), 2 to 10 (purple box), 10 to 50 (cyan box), 50 to 100 (orange circle), and more than one hundred proteins (red box), respectively. The values were plotted as histogram of frequencies with a log scale in the X-axis. The same dataset and approach is used to avoid redundancy as in Fig. 2. (6.42 MB TIF) [file pcbi.1000750.s003.tif]

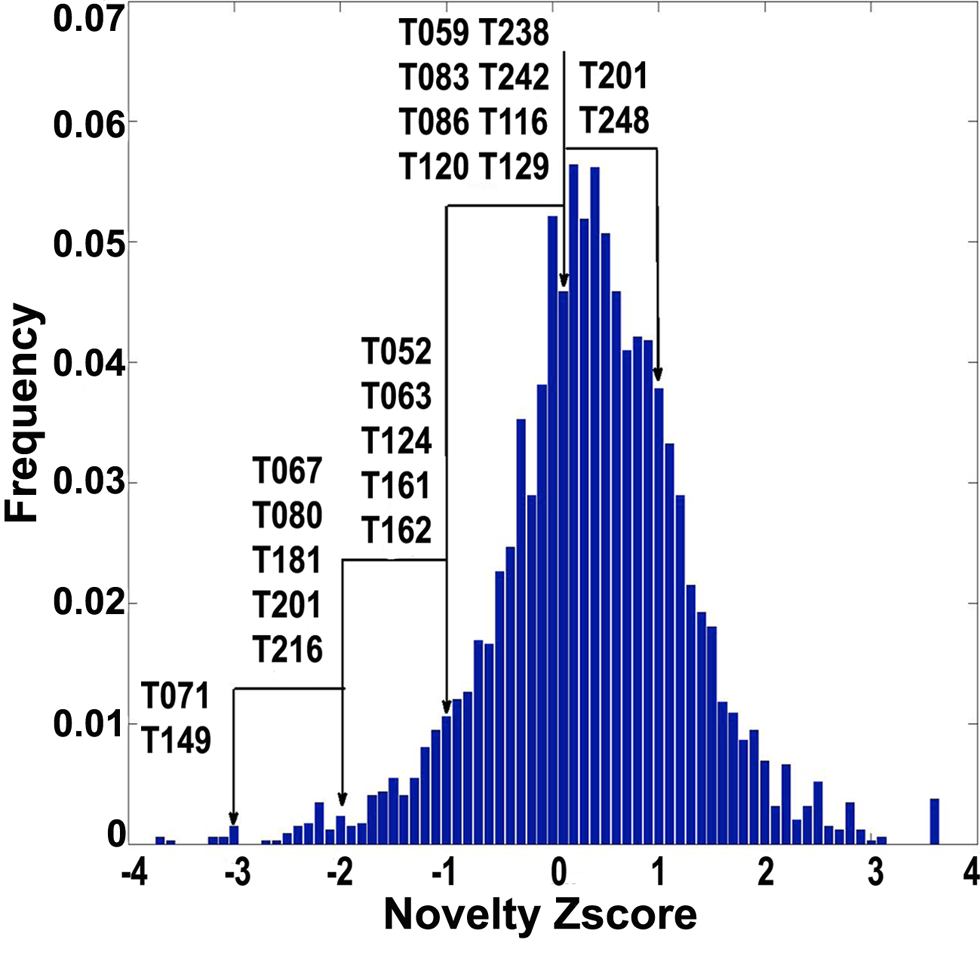

Supplement: Figure - S4 — Histogram of Novelty Z-scores of known folds in CASP dataset. Z-scores were binned by increments of 0.1. Overlaid are the Novelty Z-score for each individual new fold target submitted to CASP meetings (2.92 MB TIF) [file pcbi.1000750.s004.tif]

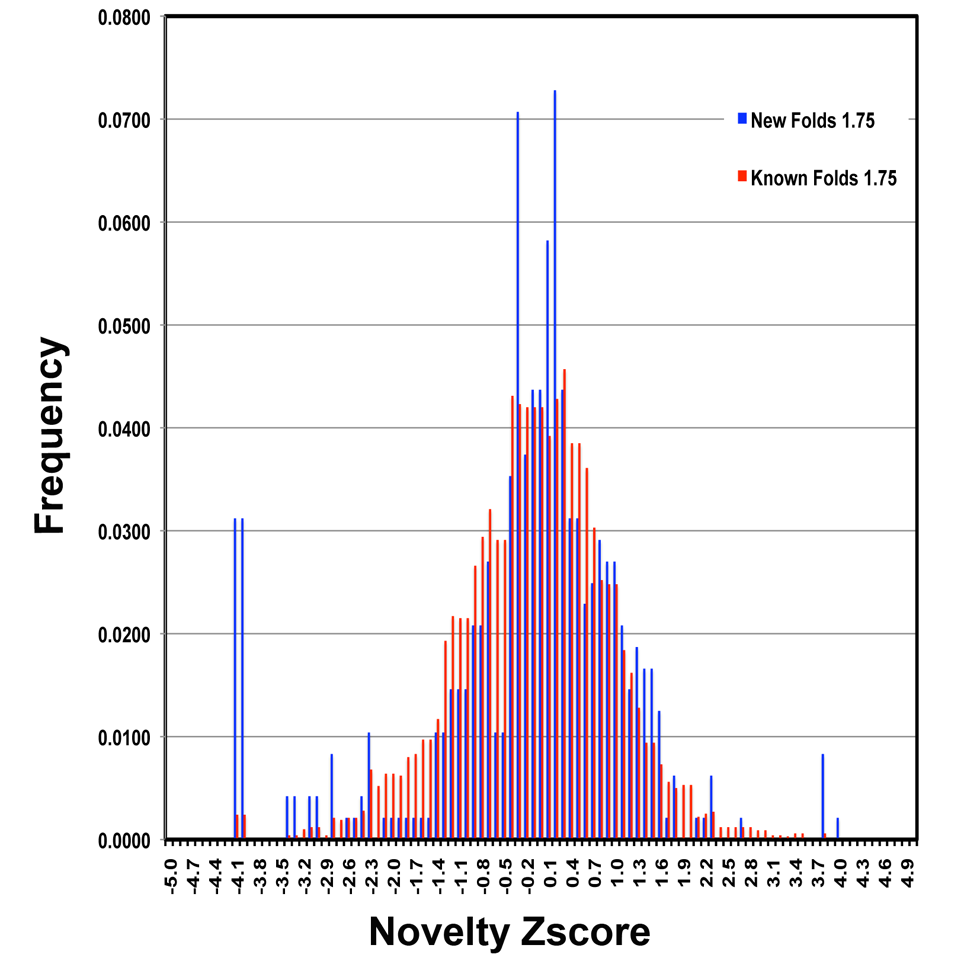

Supplement: Figure - S5 — Histogram of Novelty Z-scores of known (red) and new (blue) folds in SCOP 1.75 dataset. Z-scores were binned by increments of 0.1. (2.92 MB TIF) [file pcbi.1000750.s005.tif]

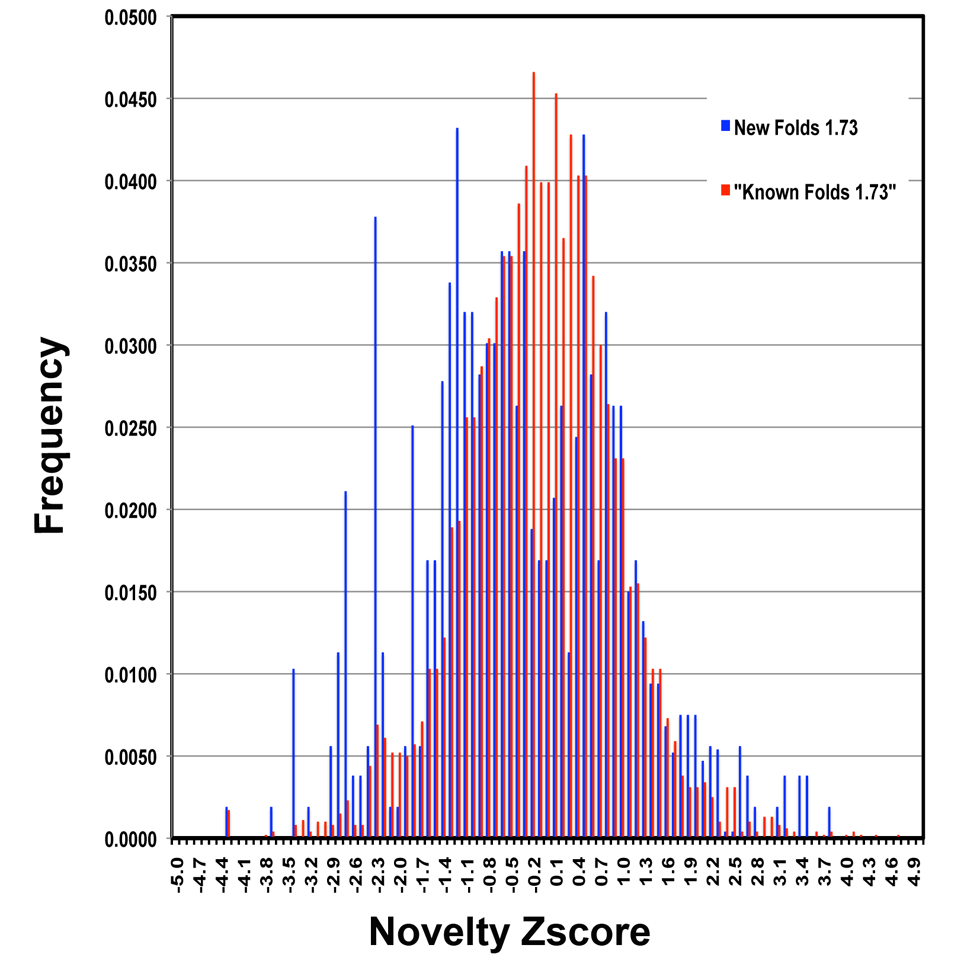

Supplement: Figure - S6 — Histogram of Novelty Z-scores of known (red) and new (blue) folds in SCOP 1.73 dataset. Z-scores were binned by increments of 0.1. (2.92 MB TIF) [file pcbi.1000750.s006.tif]

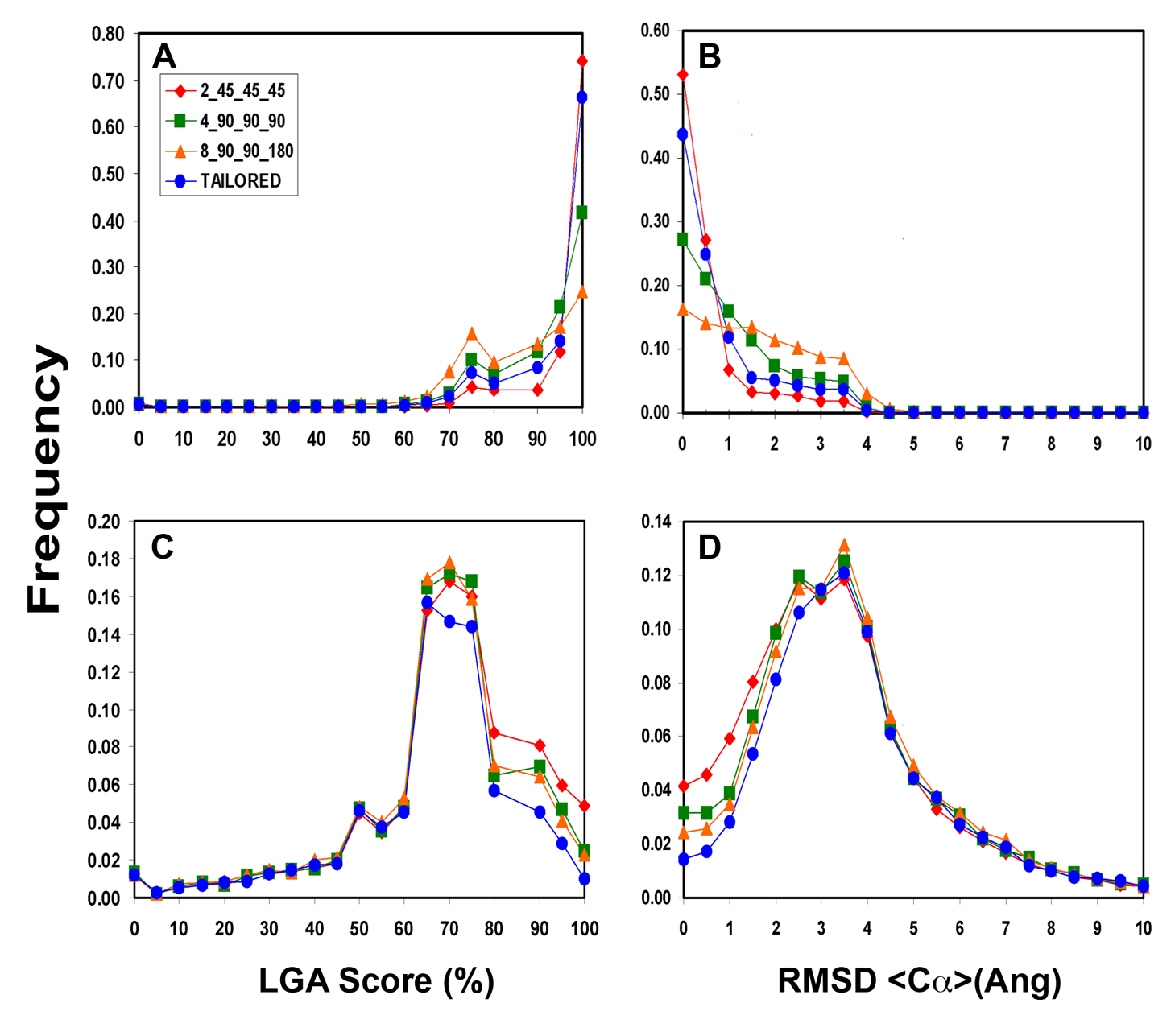

Supplement: Figure - S7 — Structural similarity vs. geometry binning. Panels A and B show the distribution of LGA score [40] and RMSD (Cα) of pairs of Smotifs that share the same geometry bin for different of bin definition: red rhomboid: 2_45_45_45, blue circle: tailored binning (see Materials and Methods section), green square: 4_90_90_90, orange triangle: 8_90_90_180; where for instance the binning 2_45_45_45 means that D in binned in interval of 2Å, and δ, θ, and ρ angles in 45 degrees respectively. Panels C and D are analogous to A and B but result from the comparison of pairs of Smotifs that have different geometries. (5.67 MB TIF) [file pcbi.1000750.s007.tif]
